# Biased Pol II fidelity contributes to conservation of functional domains in the *Potato spindle tuber viroid* genome

**DOI:** 10.1371/journal.ppat.1009144

**Published:** 2020-12-22

**Authors:** Jian Wu, David M. Bisaro

**Affiliations:** Department of Molecular Genetics, Center for Applied Plant Sciences, Center for RNA Biology, and Infectious Diseases Institute, The Ohio State University, Columbus, Ohio, United States of America; Institute of Microbiology, CHINA

## Abstract

Accurate calculation of mutation rates for viruses and viroids is necessary for evolutionary studies and to evaluate adaptation potential. However, estimation of *in vivo* mutation rates is complicated by selection, which leads to loss or proliferation of certain mutations. To minimize this concern, lethal mutations, including nonsense and non-synonymous mutations, have been used to determine mutation rates for several viruses and viroids, including *Potato spindle tuber viroid (PSTVd)*. However, this approach has limitations, including focus on a relatively small number of genome sites and the possibility that mutations may not actually be lethal or may be maintained by wild type individuals. To avoid selection bias altogether, we sequenced minus-strand PSTVd dimers from concatemeric replication intermediates. The underlying rationale is that mutations found in only one of the monomers were likely generated *de novo* during RNA polymerase II (Pol II) transcription of the circular plus-strand RNA genome. This approach yielded an apparent Pol II error rate of ~1/1837 nucleotides per transcription cycle, and an estimated mutation rate of ~1/919 nucleotides for a single replication cycle. Remarkably, *de novo* mutations were nearly absent from the most conserved, replication-critical regions of the PSTVd genome, suggesting that sequence conservation is a consequence of both essential function and template optimization for greater Pol II fidelity. Such biased fidelity may constitute a novel strategy to ensure population success while allowing abundant sampling of sequence space in other genome regions. Comparison with variants in progeny populations derived from a cloned, wild type PSTVd master sequence revealed that most *de novo* mutations were lost through selection.

## Author summary

Polymerase errors are the major source of variation in virus and viroid genomes, and as a consequence polymerase error rates are major determinants of adaptation potential. Accurate calculation of *in vivo* mutation rates is complicated by selection. To circumvent this issue, dimeric PSTVd minus-strand replication intermediates generated *in vivo* by host RNA polymerase II (Pol II) were sequenced to identify *de novo* mutations. This analysis revealed a very high error rate for Pol II transcribing genomic PSTVd RNA, leading to an extremely high mutation rate. Remarkably, however, *de novo* mutations were rare in the most highly conserved, replication-critical genome regions, suggesting these sequences are selected for both function and enhanced transcription fidelity. This biased fidelity may reveal a novel strategy to ensure population survival while maximizing adaptation potential. Further, comparison of mutations identified by minus-strand dimer sequencing with mutations observed in progeny variants derived from wild type PSTVd showed that most *de novo* mutations were lost through selection.

## Introduction

Accurate calculation of mutation rate is crucial for evolutionary studies and, in the context of viruses and viroids, is necessary to evaluate adaptation potential and the probability that mutations conferring expanded host range, immune evasion, increased virulence, or escape from human-devised resistance strategies may arise. However, selection pressure often results in loss or proliferation of individuals with certain mutations, complicating estimation of *in vivo* mutation rates. Lethal mutations, such as nonsense and non-synonymous mutations, have been used to determine mutation rates for several viruses, the principle being that once a lethal mutation has occurred, proliferation of that genome will cease [1–4]. Because lethal mutations are unlikely to be propagated, their frequency should equal the mutation rate [1]. However, this approach limits analysis to a relatively small number of genome sites, and polymerase errors may not occur at a uniform frequency genome-wide. Further, lethal mutations have not been functionally confirmed in many cases, and it is also possible that some could be maintained in progeny populations by wild type individuals, leading to biased results [5].

**Fig 1:**
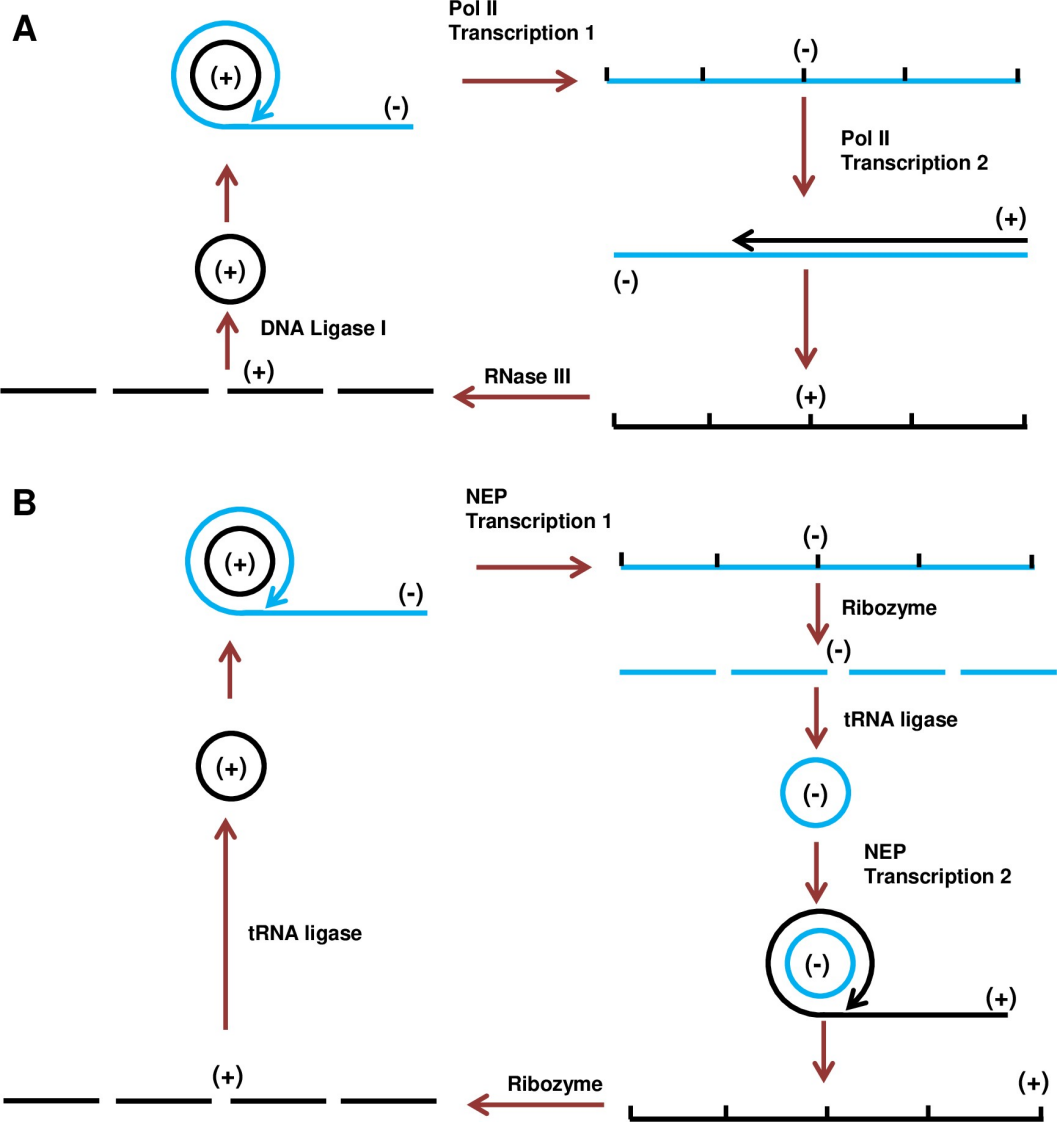
Rolling cycle replication of viroids. (A) Asymmetric rolling circle replication in the *Pospiviroidae* occurs in the nucleus. Circular (+)-strand genomic RNA is transcribed by DNA-dependent RNA polymerase II (Pol II) to produce linear (-)-strand concatemers, which serve as template for Pol II-catalyzed synthesis of linear (+)-strand concatemers. These are cleaved to unit-length monomers by RNase III and circularized by DNA Ligase I. (B) Symmetric rolling circle replication in the *Avsunviroidae* occurs in the chloroplast. Circular (+)-strand genomic RNA is transcribed by nuclear-encoded chloroplast RNA polymerase (NEP) to generate linear (-)-strand concatemers, which are cleaved to monomers by an encoded hammerhead ribozyme and circularized by a tRNA ligase. Circular (-)-strand monomers serve as template for NEP-catalyzed synthesis of linear (+)-strand concatemers, followed by a second ribozyme cleavage to form monomers, which are circularized by tRNA ligase.

We research the biology of *Potato spindle tuber viroid* (PSTVd). PSTVd has a circular non-coding RNA genome of 359 nucleotides that replicates and spreads systemically in host plants. Thus, all functions needed to establish a productive infection are mediated by sequence and structure elements within the genomic RNA [6]. Viroid species are placed in two distinct families, the *Pospiviroidae* (type member PSTVd) and the *Avsunviroidae* (type member *Avocado sunblotch viroid*) [6–10]. Genome amplification in both families occurs by rolling cycle replication (RCR). Pospiviroids replicate in the host cell nucleus by a mechanism described as asymmetric (Fig 1A). Host DNA-dependent RNA polymerase II (Pol II) transcribes the monomeric circular, genomic RNA (plus-strand) to generate linear minus-strand concatemers that serve as replicative intermediates [11–14]. (Hereafter (+)-strand and (-)-strand). With (-)-strand concatemers as template, Pol II synthesizes linear (+)-strand concatemers. After entering the nucleolus [15], these are specifically cleaved to unit-length by an RNase III activity, and the resulting monomers are circularized by DNA ligase I to form the mature genome [16–18]. The circular (+)-strand genome monomer is by far the predominant form of PSTVd RNA in infected cells. By contrast, members of the *Avsunviroidae* replicate in the chloroplast through a symmetric mechanism (Fig 1B). The nuclear-encoded chloroplast RNA polymerase (NEP) transcribes the genome to produce (-)-strand concatemers, which are co-transcriptionally cleaved to monomers by an encoded hammerhead ribozyme and circularized by a tRNA ligase [19–21]. Circular (-)-stand monomers then serve as template for synthesis of linear (+)-strand concatemers, which are similarly cleaved by an embedded hammerhead ribozyme and circularized to generate mature viroid genomes [22].

For viruses and viroids, mutation rate is largely determined by the rate at which errors are generated during genome replication [23]. Mutation rates for double-stranded DNA viruses typically range between ~10^−6^ to 10^−8^ substitutions per nucleotide per cell infection, whereas most RNA virus rates vary between 10^−4^ to 10^−6^ [5,23–25]. The higher mutation rates of RNA viruses are attributed in part to the error-prone nature of viral RNA-dependent RNA polymerases, which (with the exception of Coronaviruses) lack proofreading activity [26]. Following this reasoning, analysis of viroid mutation rates should provide insight into the error rates of host DNA-dependent RNA polymerases when co-opted to transcribe non-native RNA templates. Two previous studies used the lethal mutation method to show that viroids, which have the simplest genomes among all biological systems, exhibit the highest mutation rates. Examining the catalytic core nucleotides of (+)- and (-)-strand hammerhead ribozymes (total 32 sites) from RT-PCR clones, a remarkably high mutation rate of 1/400 nucleotides, approaching one mutation per genome, was reported for *Chrysanthemum chlorotic mottle viroid* (CChMVd, family *Avsunviroid*ae) [27]. Using similar hammerhead sites in conjunction with next generation duplex sequencing, a second study calculated that the spontaneous mutation rate of another chloroplast-replicating viroid, *Eggplant latent viroid* (ELVd), ranged from 1/800 to 1/1000 [28]. Together these estimates suggest that chloroplast NEP transcribes RNA templates with extremely low fidelity. The second study also included the nuclear-replicating PSTVd, which lacks ribozyme sequences. In this case, analysis focused on the central conserved region (CCR, ~54 nucleotides) and the terminal conserved region (TCR, ~16 nucleotides), where mutations are expected to have detrimental or lethal outcomes [6, 29]. An additional 23 sites where mutations resulting in impaired infectivity or lethality have been reported were also included [30–34]. From these sequences, the PSTVd mutation rate was estimated to range between 1/3800 to 1/7000 nucleotides, suggesting that Pol II also transcribes RNA templates with low fidelity, although much more accurately than NEP [28]. A shortcoming of these studies, imposed by the lethal mutation method, is a focus on a small number of conserved sites. As mutation clusters have been observed in viroid RNA [35], it is unclear whether results obtained from a limited number of lethal mutations are representative of the entire genome. Further, some conserved regions (e.g. the CCR and TCR) are likely to tolerate mutations. Therefore, an unbiased method to calculate the PSTVd mutation rate at the whole genome level was needed.

Inspired by the existence of (-)-strand concatemers in PSTVd infected cells, we reasoned that by cloning and sequencing portions of these (dimers), mutations shared or not shared by two monomers of the same dimer can be identified. Shared mutations would most likely be derived from genomic (+)-strand RNA templates, while unshared mutations would most likely be generated *de novo* during transcription of an individual monomer by Pol II. This approach allows the full spectrum of mutations (polymerase errors, including base substitutions, insertions, and deletions) to be identified across the entire genome without complications arising from selection. Using this method, we obtained an apparent error rate for Pol II transcribing PSTVd RNA that is considerably greater, by approximately two orders of magnitude, than estimated error rates for Pol II transcribing mRNAs from DNA templates. The error rate was extrapolated to infer a PSTVd mutation rate for a single replication cycle that is similar to mutation rate estimates for the chloroplast-replicating viroids. When mapped at the whole genome level, *de novo* mutations were not evenly distributed and were largely absent from the most highly conserved regions, suggesting that these regions reflect selection for greater Pol II fidelity in addition to critical function. Moreover, comparison with natural variants observed by deep sequencing progeny derived from a cloned PSTVd master sequence indicated that most *de novo* mutations were lost through selection.

## Results and discussion

### PSTVd (-)-strand concatemers contain up to seven copies of the unit-length genome

**Fig 2:**
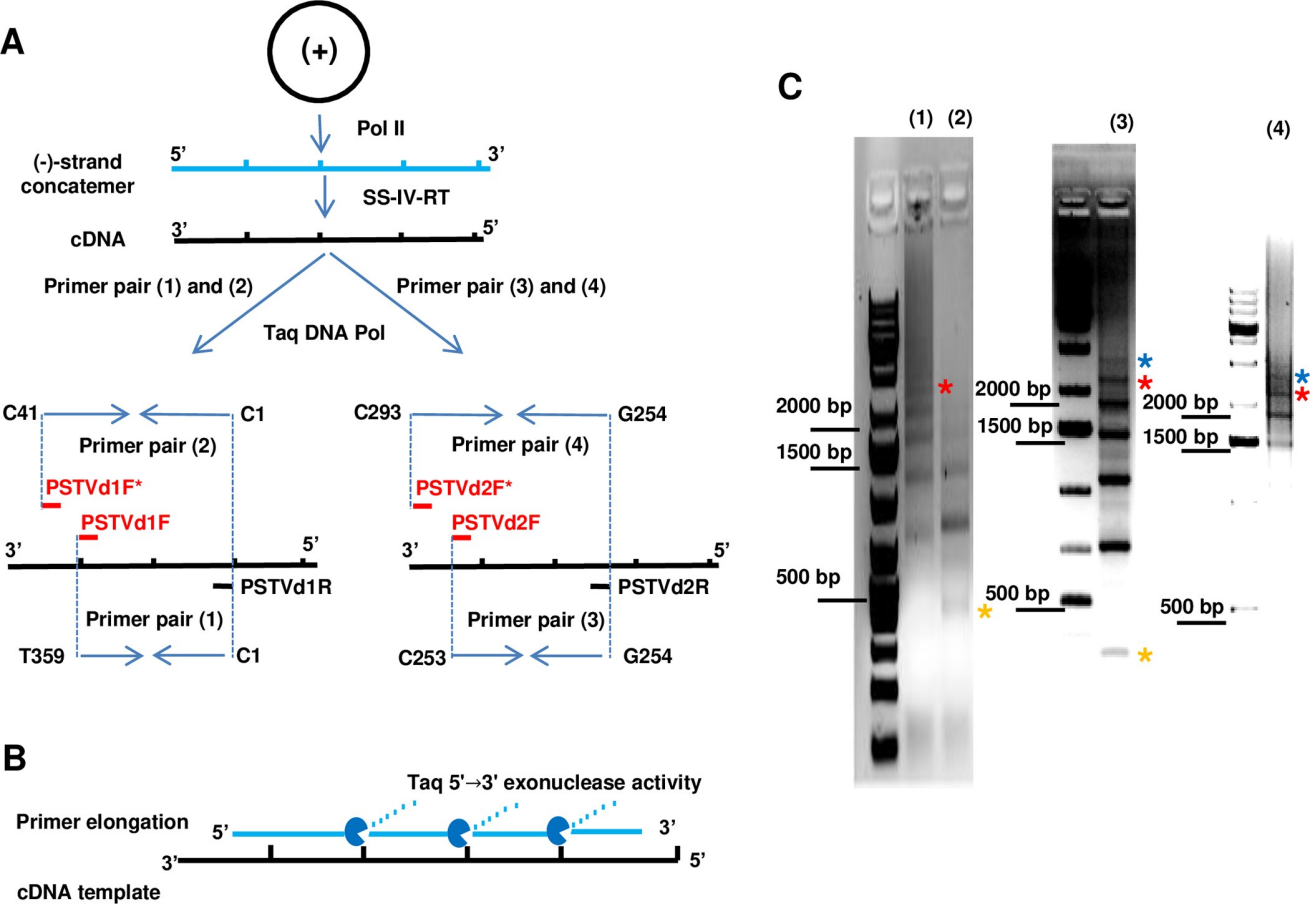
PCR amplification of (-)-strand PSTVd concatemers. (A) Schematic diagram shows transcription of (+)-strand PSTVd monomer by Pol II to synthesize linear (-)-strand concatemer, followed by cDNA synthesis from (-)-strand RNA using Superscript IV reverse transcriptase (SS-IV-RT). Taq DNA polymerase was employed to perform PCR amplification of duplex DNA copies from the cDNA using four separate primer pairs, as shown. The nucleotide coordinates of forward (F) and reverse (R) primers are indicated. Primers can bind to the same sites in all monomers, but for simplification only one binding site is indicated for each. PCR reactions (1) and (3) yield products with primers included in the concatemers, while reactions (2) and (4) yield products with concatemers flanked by primers (indicated by an asterisk). (B) With 5'→3' exonuclease activity, Taq DNA polymerase is able to synthesize through downstream products (nick translation). (C) PCR products amplified by Taq DNA Polymerase using primer sets 1 through 4 were resolved by 1% agarose gel electrophoresis. Ladders of discrete products correspond to genome concatemers. Monomer, hexamer, and heptamer products are indicated by asterisks (yellow, red, and blue, respectively).

It is well-established that Pol II transcribes monomeric (+)-strand PSTVd RNA genomes by RCR to generate linear, concatemeric (-)-strand replicative intermediates [36]. For the purposes of this study, a rough estimate of the size range obtainable by PCR was needed. To approach this question, it was assumed that the activity of Superscript IV reverse transcriptase (SS-IV-RT), which has strand displacement activity and is capable of copying the ~9 kilobase (kb) genome of Molony murine leukemia virus (MMLV) from which it is derived, is not a limiting factor. We also took advantage of the fact that Taq DNA polymerase, which can generate products up to 10 kb in length, has 5'→3' exonuclease activity. A diagram of the protocol is presented in Fig 2A. First, cDNA was prepared using RNA obtained from combined upper leaves of *Nicotiana benthamiana* plants harvested two- and three-weeks after inoculation with PSTVd. Reverse transcription was carried out with primers specific for (-)-strand PSTVd RNA. Using this cDNA as template, duplex DNA copies were amplified using Taq polymerase. Separate PCR reactions were performed with four different primer sets, with each set capable of generating unit-length monomers and multiples thereof. All of the primers could recognize multiple binding sites on a concatemeric template. However, 5'→3' exonuclease activity allows Taq to remove downstream products and continue synthesis (equivalent to nick translation), albeit likely with a reduced rate and processivity (Fig 2B). Following gel electrophoresis, all four PCR primer pairs yielded a ladder of discrete products corresponding to genome multimers, with product abundance decreasing with increasing size. Primer pairs 3 and 4 yielded the largest PCR products clearly resolved in the gel (>2 kb), which appear to be heptamers (359 nt x 7 = 2513) (Fig 2C). Larger material, some of which might represent even higher-order multimers, is also evident as a smear in the gels. The limitations of this experimental protocol are several, and include reduced activity of Taq polymerase when performing nick translation and the relatively low resolving power of the gel system employed. Nevertheless, while it was not our intention to determine the largest possible number, it is apparent that Pol II can transcribe circular (+)-strand PSTVd into (-)-strand concatemers that contain up to at least seven monomers, which should be regarded as a minimum estimate. Our results are in agreement with previous RNA blot hybridization studies, which detected (-)-strand PSTVd concatemers comprised of up to six and seven monomers [37,38].

### Dimer sequencing reveals a very high error rate for Pol II transcribing PSTVd RNA

As noted in the Introduction (Fig 1), members of the *Avsunviroidae* generate RCR intermediates that are co-transcriptionally processed to monomers, whereas concatemeric (-)-strand PSTVd replication intermediates can be amplified from infected plants (Fig 2C). Thus, PSTVd was used as a model to evaluate the dimer-sequencing method for calculation of viroid mutation rates. Because (+)-strand concatemers are the result of two independent transcription events, (-)-strand dimer sequencing was chosen in order to analyze Pol II error rate in a single transcription cycle. In addition, there was concern that genome monomers would reduce the chances of obtaining the desired dimers from PCR reactions. Complete removal of circular (+)-strand genome monomers, the most abundant form of PSTVd RNA in infected cells, is technically challenging, while circular (-)-strand monomers are typically not detected [37,38].

**Fig 3:**
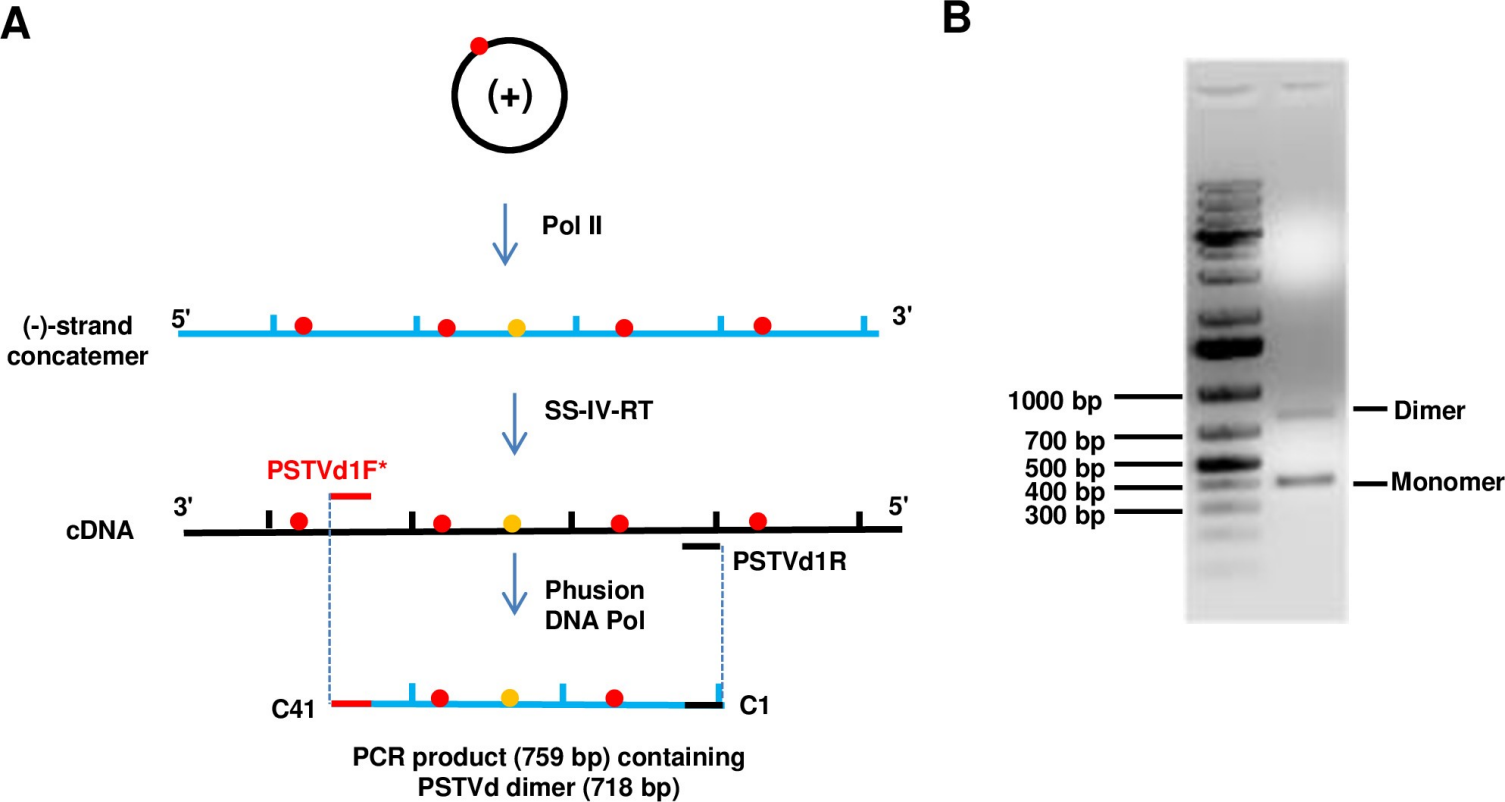
PCR amplification of (-)-strand PSTVd dimers and identification of *de novo* mutations. (A) Diagram showing Pol II transcription of (+)-strand PSTVd monomer to (-)-strand linear concatemer, cDNA synthesis from (-)-strand RNA by Superscript IV reverse transcriptase (SS-IV-RT), and PCR amplification of dimeric duplex DNA from the cDNA by Phusion DNA polymerase. Shared and unshared mutations in individual monomers are represented by red and yellow dots, respectively. Primer pair 2 (see Fig 2) was used to amplify PCR products containing PSTVd monomers and dimers. (B) Monomer and dimer products were separated by 1% agarose gel electrophoresis. Dimer bands were excised from the gel, purified, and cloned.

An overview of the experimental protocol is illustrated in Fig 3A. Three independent experiments were performed, with four plants each. These were designated replicates A, B, and C (RA, RB, and RC). Using SS-IV-RT, cDNA copies of PSTVd (-)-strand concatemers were obtained from RNA extracts prepared as before from infected *N*. *benthamiana* plants. Since reverse transcriptases are known to be error prone, this step is the most likely source of background mutations in the protocol. However, error rates reported for MMLV-based reverse transcriptases range from ~1/15,000 to 1/30,000 [39,40], which is substantially lower than the anticipated Pol II error rate on viroid RNA templates. Phusion DNA polymerase, which has the highest fidelity of currently available PCR enzymes (error rate 10^−6^ to 10^−7^), was used to amplify duplex DNA copies from the cDNA. A single primer pair (pair 2, see Fig 2 and Materials and methods) was employed. Because Phusion polymerase lacks 5'→3' exonuclease activity, nick translation is not possible. As a result, only monomer and dimer products were obtained, with monomer being most abundant (Fig 3B). Dimer bands were excised from the gel, and extracted DNA was cloned and Sanger sequenced. In a control experiment, a cloned PSTVd dimer was transcribed *in vitro* using T3 RNA polymerase, and transcripts were subjected to the same reverse transcription and PCR steps using the same enzymes and primers.

From the three biological replicates 91, 86, and 84 dimer clones were sequenced (Table 1). As each PSTVd dimer is 718 nucleotides in length, this corresponds to 65,338; 61,748; and 60,312 sites examined for RA, RB, and RC, respectively. So, a combined total of 261 dimer clones, comprising 187,398 nucleotides and representing 522 genome equivalents, were included in subsequent analyses. Each sequenced dimer was examined for mutations found in only one of the monomer units (i.e. unshared *de novo* mutations) (Fig 3A). From this analysis, 30 mutations (12 base substitutions, 10 insertions, and 8 deletions), 35 mutations (19 base substitutions, 10 insertions, and 6 deletions), and 37 mutations (15 base substitutions, 8 insertions, and 14 deletions) were identified for experimental replicates RA, RB, and RC, respectively, yielding Pol II error rates of 1/2178, 1/1764, and 1/1630 nucleotides per transcription cycle (Table 1). Only one shared mutation (123A124, described below) was observed in one of the replicate experiments. Across all three experiments, a total of 102 mutations were found in the 187,398 nucleotides examined, for an average of 1/1837 nucleotides per transcription cycle (apparent error rate ~5.4 x 10^−4^ per nucleotide per transcription cycle). In the control experiment beginning with a (-)-strand PSTVd dimer transcribed *in vitro* with T3 RNA polymerase, a total of 86 dimer clones (61,748 nucleotides) were sequenced and only one unshared *de novo* mutation (A50G) was found. This base substitution was not identified in the collection of unshared mutations from the three experimental replicates.

**Table 1:** Frequency and type of unshared *de novo* mutations observed in PSTVd (-)-strand dimer clones.

	RA	RB	RC	Total (RA, RB and RC)	Control
Number of dimers	91	86	84	261	86
Number of nucleotides examined	65,338	61,748	60,312	187,398	61,748
Mutations:					
Insertions	10	10	8	28	
Deletions	8	6	14	28	
Substitutions	12	19	15	46	1
Total mutations	30	35	37	102	1
Pol II error rate (nucleotides per transcription cycle)	1/2178	1/1764	1/1630	1/1837	
Mutation rate* (nucleotides per replication cycle)	1/1089	1/882	1/815	1/919	

Three independent experiments, RA, RB, and RC, were performed. For each biological replicate, the number of dimers sequenced, the number of total PSTVd nucleotides examined, and the number and type of *de novo* (unshared) mutations observed are indicated. Apparent Pol II error rates were calculated from these data. Results of a control experiment done with an *in vitro* transcribed (-)-strand PSTVd dimer are also shown.

*Mutation rates were extrapolated from error rates. See text for details.

In infected plants, individual (-)-strand concatemers likely serve as template for multiple (+)-strand concatemers, each of which can generate multiple PSTVd progeny genomes that could infect new cells. To extrapolate a mutation rate for a single replication cycle, it was assumed that this second transcription step (namely (-)-strand concatemer to (+)-strand concatemer) occurs with an error rate similar to the first. Unfortunately, we could not obtain direct evidence to support this assumption, as it was not technically feasible to use the dimer sequencing protocol in this case. PSTVd (+)-strand concatemers accumulate to very much lower levels than (-)-strand concatemers in infected cells while the opposite is true of monomer forms [38], rendering amplification of (+)-strand dimers exceedingly difficult. In any case, while polymerase fidelity can vary somewhat depending on template sequence and structure, expectation of a roughly similar overall error rate is not unreasonable. Thus, an average mutation rate of ~1/919 nucleotides was calculated for a single replication cycle, assuming twice as many mutations would occur over the same number of total nucleotides examined (i.e. 204/187,398). We suggest that this method of analysis provides an estimate of potential mutation rate based on polymerase error frequency, and expect the rate and mutation spectrum to be impacted by selection.

This estimate (1/919) is greater than the PSTVd mutation rate of 1/3900 to 1/7000 reported previously [28], and more similar to mutation rates determined for the chloroplast-replicating viroids CChMVd and ELVd [27,28]. Plausible explanations for the varied PSTVd estimates are several and include differences in experimental approach, host plants, and infection period. Our estimate is based on Pol II error rate over a single replication cycle using tissue obtained two to three weeks post-inoculation of *N*. *benthamiana* plants. The earlier PSTVd study applied the lethal mutation method to progeny populations with tissue obtained and 6- and 18 months post-inoculation of eggplant, over which time an unknown number of replication cycles would have occurred. Thus, direct comparisons are difficult. In any event, mutation rates of the magnitude observed in our study are likely sustainable given the small size of the PSTVd genome and could enhance population survival by providing abundant variation for selection.

Major advantages of the (-)-strand dimer sequencing method are that it allows the entire PSTVd genome to be surveyed in the absence of selection bias. However, it should be pointed out that the protocol will not detect misincorporation events that lead to chain termination, and so the error rate derived from these experiments should be regarded as an under-estimate. Most probably such events are rare and unlikely to have a significant impact on overall findings. Nevertheless, from sequencing 522 genome equivalents over three independent experiments, we observed an apparent error rate of ~5.4 x 10^−4^ per nucleotide per transcription cycle for Pol II transcribing PSTVd RNA. Judging by the detection of one unshared mutation in the control experiment, which covered 172 genome equivalents, the background mutation rate was negligible. This perhaps is not surprising, considering the error rate of SS-IV-RT is about 10-fold lower (~3 to 6 x 10^−5^ vs. ~5.4 x 10^−4^). It has been reported that in *Caenorhabditis elegans* and *Saccharomyces cerevisiae*, Pol II transcribes mRNAs from DNA templates with an error rate of ~3.9 to 4.0 x 10^−6^ per base pair [41,42], and a similar error rate might be expected for the *N*. *benthamiana* transcription machinery. Clearly, Pol II transcribes PSTVd RNA with much lower fidelity (~100-fold). An obvious explanation for the dramatically increased error rate is that RNA is not the native template, which might impact nucleotide selectivity. Other possibilities are that the function of components which normally enhance Pol II fidelity, such as subunit RPB9 and transcription factor IIS (TFIIS), are impaired [43]. Moreover, pathways that eliminate defective mRNAs, like nonsense-mediated decay [44], would have no role in improving the quality of transcription products generated from an unspliced, non-coding viroid RNA. Another consideration is the involvement of a non-canonical co-factor that likely mediates use of an RNA template. Synthesis of PSTVd (-)-strand concatemers by Pol II *in vitro* is facilitated by a plant-specific, seven-zinc finger splice variant of transcription factor IIIA (TFIIIA-7ZF) [14,45]. TFIIIA is a general transcription factor that normally functions with RNA polymerase III. How TFIIIA-7ZF might influence Pol II template preference, and if PSTVd transcription involves additional non-native co-factors *in vivo*, is not known.

### The spectrum of Pol II transcription errors on PSTVd RNA template

Of the 261 sequenced dimer clones, 77 contained a *de novo* mutation. While the majority of these (60) had only one mutation, 12 had two mutations, four had three mutations, and one had six individual mutations (total of 102, Table 2). All but one of the mutations involved a single base substitution, insertion, or deletion. The exception was a deletion of seven consecutive bases found in replicate A, which was scored as a single mutation.

**Table 2:** Unshared *de novo* mutations identified in PSTVd (-)-strand dimers.

RA	RB	RC
123A124	10A11/G342C/U331G	123A124
G54A	199G200	60A61/C189U
U332C	123A124/U340C/346G347	Δ34C/Δ41C/Δ180U/Δ300U/Δ309U/Δ322G
123A124	G61A/A63G	G322A
Δ57-AAAAGAA-63	U24C/C301U	Δ180U
123A124	53A54	C224U
C127U	Δ180U	123A124
Δ180U	A290U/A334U/A170U	A53G
A275G	123A124	G116A/G217A/G246A
123A124	123A124	123A124
G322A	123A124	96U97
C208U	U252A	123A124/C338A
Δ180U	C303U/123A124	G124A
C208U	G249A	A111C
96U97	G294A	G217A
123A124	123A124	115U116
Δ204C	G75A	Δ246G
C127U	Δ31C	Δ253C
C259U/Δ310A	Δ350G	U157A/U202C
C181U	G49A/Δ180U	Δ7U
C216A	C204U/G249A	123A124
Δ310A	G131U/Δ311C	Δ10A
123A124	Δ11C	C256U/Δ7U
Δ41C		G124A
60A61/123A124		Δ305A
123A124		Δ310A
G289A		
Δ41C		

Unshared *de novo* mutations found in dimer clones from the three replicate experiments are shown. The 102 total *de novo* mutations included 46 base substitutions and 56 indels. As some mutations appeared in multiple genomes, only 64 were unique. Bases involved and genome coordinates are given for each mutation type: base substitutions (e.g. G322A), single base insertions (e.g. 123A124), and single base deletions (e.g. Δ180U). Only one mutation involved more than one base (Δ57-AAAAGAA-63). Mutations observed in more than one replicate are indicated in red. Two mutations, 123A124 and Δ180U, were found in all three replicates.

**Fig 4:**
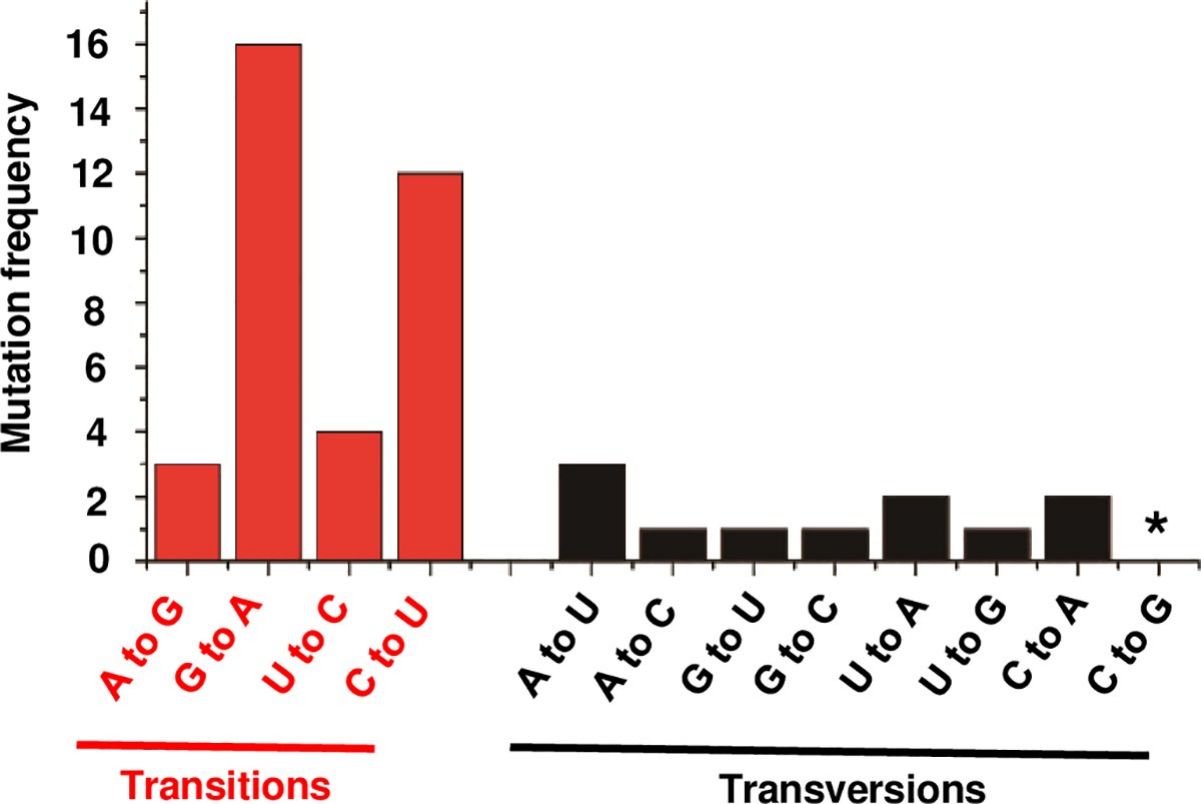
Base substitutions introduced by Pol II in PSTVd. Mutations were mapped to genomic (+)-strand PSTVd. Number of transition and transversion events observed by dimer sequencing across all three biological replicates are shown. Asterisk indicates none observed.

Among the 102 *de novo* mutations observed only 64 were unique, as 13 mutations appeared multiple times. Base substitutions accounted for nearly half of the total mutations (46/102). Of these, transitions were most common (35/46), with G→A and C→U comprising more than half of total base substitutions (Fig 4). Yeast and *C*. *elegans* Pol II are also more likely to commit transition than transversion errors on DNA templates [41,42]. Other transitions (A→G and U→C) were observed three and four times, respectively. The most abundant transversion, A→U, was also seen three times. Of the remaining seven possible transversion types, two were observed twice, four only once, and one (C→G) was not detected, suggesting that Pol II commits these substitution errors less frequently. It is interesting to consider that the possibility for G-U pairing in RNA might account for higher levels of G→A and C→U transitions observed in this analysis. However, Pol II most commonly makes these same transitions when transcribing DNA [42].

**Fig 5:**
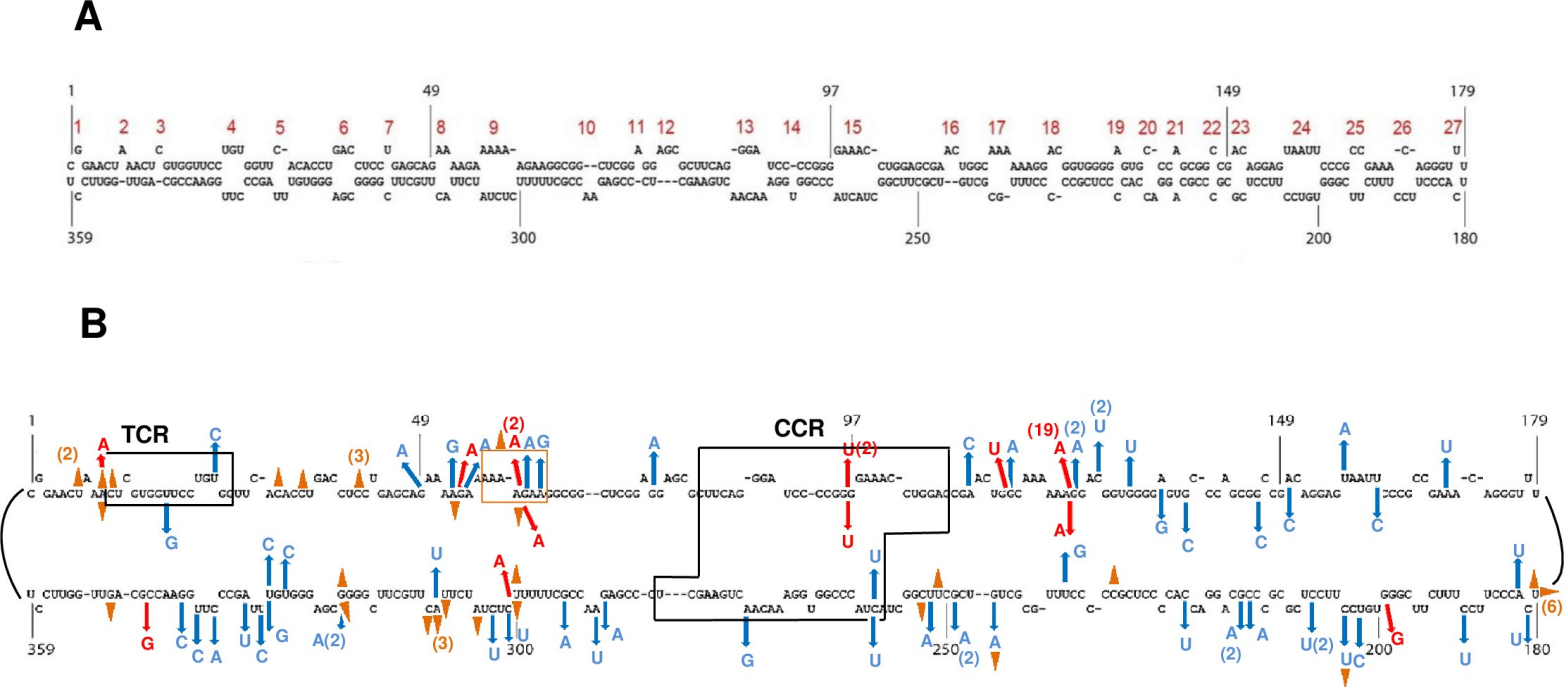
Comparison of mutations identified from PSTVd (-)-strand dimer sequencing and deep sequencing variant progeny. (A) Secondary structure of the (+)-strand PSTVd genome. Nucleotide coordinates are indicated and loops/bulges are numbered 1 to 27 (red). (B) Mutations were mapped to genomic (+)-strand PSTVd. Mutations identified from dimer sequencing are indicated to the outside of the PSTVd secondary structure, and mutations identified by deep sequencing PSTVd progeny populations are indicated to the inside. Progeny mutations shown were shared by more than 10 unique sequences per million reads. Blue arrows indicate base substitutions, red arrows indicate base insertions, and brown triangles indicate base deletions. A deletion of seven consecutive bases is indicated by a brown box. Numbers of occurrences are indicated in parentheses. The terminal conserved region (TCR) and central conserved region (CCR) are outlined by black boxes.

Small insertions and deletions (indels) comprised the remainder of the mutations (56/102). Single base insertions made up ~27% of the total mutations (28/102). Among these, an A insertion within a tract of six A residues between nucleotides 118 and 123 (5'→3' 118-AAAAAA-123) occurred 19 times (Table 2 and Fig 5). This mutation was designated 123A124. (Note: Because the insertion could be anywhere within or flanking the tract of A residues, it was arbitrarily placed after the last base in the run. The same holds for other insertions and deletions where the exact site is ambiguous.) Insertion 123A124 was one of two mutations found in all three biological replicates. This, and the fact that 123A124 sometimes appeared along with different mutations, indicates that its abundance is not an artifact of sibling clones. Four additional insertions also occurred within runs of identical residues, including 10A11 (5'→3' 7-AAA-10) 53A54 (5'→3' 50-AAAA-53), 60A61 (5'→3' 55-AAAAAA-60) which occurred twice, and 199G200 (5'→3' 197-GGG-199). Thus, 24 of the 28 total single base insertion errors were at purine homonucleotide tracts in the template RNA.

Deletions comprised about 27% of the total mutations (28/102), and these also occurred mostly within homonucleotide tracts in the RNA template. One deletion, Δ180U, which was detected six times and was present in all three biological replicates, occurred within a stretch of four U resides between nucleotides 177 and 180 (5'→3' 177-UUUU-180) (Table 2 and Fig 5). Others were observed within runs of three (Δ10A), four (Δ322G) or five (Δ300U) identical nucleotides, while four deletions (Δ34C, Δ204C, Δ309U, and Δ41C, which was detected three times) occurred within dinucleotides. Thus 14 of 27 single base deletion errors occurred within homonucleotide tracts in the template RNA. An unusual deletion, the only mutation that involved more than one base, removed seven consecutive nucleotides (Δ57-AAAAGAA-63) within a longer tract of A residues. However, we cannot rule out the possibility that this unusual deletion may be a cloning artifact.

In summary, base substitutions and indels were about equally represented in the *de novo* mutations. Further, Pol II appears more likely to commit transition errors than transversions, and preferentially commits indel errors at dinucleotide or longer homonucleotide tracts in the template RNA. Hotspots for insertion (5'→3' 118-AAAAAA-123; 123A124) and deletion (5'→3' 177-UUUU-180; Δ180U) were apparent, although why these sites might be particularly problematic is not clear. While yeast and *C*. *elegans* Pol II commit indel errors less frequently than base substitutions when transcribing mRNAs, most indels also occur at homonucleotide tracts [41,42]. This, and a common predilection for transitions over transversions, implies a similar Pol II error spectrum on DNA and RNA templates. Thus, regardless of template, Pol II is similar to DNA polymerases in more frequently committing substitution errors of the same structural class and indel errors at homonucleotide tracts [46,47].

### Transcription errors cluster in distinct regions

The PSTVd RNA genome folds into a secondary structure that contains 26 base-paired stems and 27 loops or bulges (Fig 5A). However, most loops in RNA molecules have three-dimensional (3D) structures stabilized by non-canonical base pairs and other base interactions [48,49]. PSTVd RNA is no exception, and specific functions have been ascribed to many of the structured loops [31–34,50,51].

When mapped to the (+)-strand secondary structure of the PSTVd genome, unshared *de novo* mutations (polymerase errors) were observed throughout and affected both stems (secondary structure) and loops (3D structure) (Fig 5B). However, mutations were not evenly distributed but appeared to cluster in a few distinct regions. Nucleotides 50–65 and complementary nucleotides 295–310 that encompass loop 9 suffered multiple base substitutions, insertions, and deletions that collectively impact both the loop and flanking stems. The exceptional Δ57-AAAAGAA-63 deletion also lies within this region. The stem spanning nucleotides 120–125 and adjacent loop 17 contains a tract of A residues that is the site of the major insertion hotspot (123A124). Loop 27 (nt 177–182) is the site of a deletion hotspot (Δ180U). In addition, sequences between 330–342 suffered six base substitutions within loops 4 and 5 and adjacent stems. The specific mutations noted here are yet to be tested for their effect on PSTVd replication and/or spread. However, the mutation clusters span or are adjacent to structured loops known to be essential for PSTVd replication (loop 4) or trafficking (loop 17 and loop 27), or are required for efficient replication and trafficking (loop 9) [33,51]. That mutation clusters were observed in and around these essential sequences and structures underscores the fact that (-)-strand dimer sequencing obviates selection bias.

### Conserved regions of the PSTVd genome appear be a consequence of high Pol II fidelity

The CCR and TCR domains mediate key functions and are more conserved than other regions of the PSTVd genome [6,29] (Fig 5). The CCR contains a loop E motif (loop 15) that is essential for replication and serves as a binding site for RPL5, an apparent regulator of replication [31,52]. It also contains additional loops (13 and 14) critical for replication [33], as well as the (+)-strand cleavage and ligation site located between G95 and G96 [53]. The TCR harbors structures and sequences required for (-)-strand transcription, including loops 3 and 4 and binding sites for Pol II and TFIIIA-7ZF [14,33,54]. Together, the CCR and TCR comprise 70 nucleotides, accounting for ~20% of the genome. However, dimer sequencing identified only six *de novo* mutations within the CCR and TCR, accounting for ~6% of the 102 identified mutations (Fig 5B). Therefore, Pol II generates fewer mutations when transcribing these domains compared to other regions. This in part explains our higher single cycle mutation rate (1/919) compared to the earlier estimate (1/3900 to 1/7000) that focused mainly on the CCR and TCR [28]. Considering these domains alone, our dimer sequencing data would yield a Pol II error rate of 1/6090 nucleotides per transcription cycle (6 mutations in 36,540 nucleotides examined), corresponding to an extrapolated mutation rate of 1/3045 for a single replication cycle, much closer to the earlier estimate. This comparison underscores the advantage of scanning the entire genome, which presents a broader picture of the overall mutation rate.

A broader view can also lead to new insight. Sequence conservation is usually and rightly taken as evidence of important function. However, an evaluation of the landscape of polymerase errors across the PSTVd genome allows us to suggest that the high degree of sequence conservation within the CCR and TCR is not due solely to function-based selection, but is also a consequence of high Pol II fidelity when transcribing these regions. This stands in contrast to mutation clusters found in other genome regions known to have essential functions, as noted above. Thus, we propose that the most critical conserved regions may arise as a result of template optimization for enhanced transcriptional fidelity, suggesting co-evolution of PSTVd and the host transcription apparatus. How this might occur is unclear, and answers await further analysis of PSTVd transcription and transcription complexes. In any event, a high overall mutation rate coupled with high fidelity transcription in domains most critical for PSTVd replication and genome maturation may enhance survival while allowing maximal sampling of sequence space in important but less critical regions of the genome. It will be interesting to see if RNA virus genomes and polymerases have evolved similar strategies.

### The majority of *de novo* mutations are lost through selection

In a previous study, progeny populations generated following inoculation of *N*. *benthamiana* plants with a cloned wild type PSTVd master sequence were profiled using an approach that allowed sequencing of complete genomes [55]. PSTVd progeny populations in both inoculated leaves and systemically infected leaves (two replicates each) were deep sequenced using the MiSeq platform. For the purposes of this study, we focused on the two libraries prepared from systemically infected leaves, as progeny would likely be selected for ability to both replicate and traffic to leaves above the inoculation site. Both libraries were prepared from pooled leaves collected at 14- and 21-days post-inoculation. Individually, the two libraries contained more than 2.74 x 10^6^ and 2.78 x 10^6^ complete PSTVd sequences, respectively, including genomes with indels. Of these, 82% were wild type PSTVd and 18% were variants with at least one mutation. Variants with more than one mutation were rare (3.12%). To minimize errors resulting from library preparation, sequences with fewer than 30 reads per million were eliminated, leaving 1,094 and 1,231 unique genome sequences in subsequent analyses. Comparing the two libraries against the wild type PSTVd master sequence showed that together, variant populations included alterations at nearly all sites in the genome. Only five invariant nucleotides were identified: G175, U339, U340, A343, and C358. Interestingly, a base substitution at U340 (U340C) was observed among the *de novo* mutations.

In theory, if variants constitute ~18% of progeny populations and if all variants accumulate at levels similar to wild type, we might expect to observe ~47 (-)-strand dimer clones with a shared mutation among the 261 sequenced in the present study (~18% of 261 dimer clones). However, only a single shared mutation (123A124) was identified in one dimer, suggesting that most of the mutant genomes generated during transcription do not replicate or accumulate to a significant extent.

To compare mutations in progeny populations with *de novo* mutations, we considered those identified in 10 or more unique sequences per one million reads (above the 30 per million threshold) in either or both libraries. Only 24 unique mutations met this criterion, including 12 base substitutions, 4 single base insertions, and 8 single base deletions. These 24 unique mutations were represented by 731 total mutations (Table 3). Reflecting their stochastic nature, mutations were not always observed in replicate libraries, and individual frequencies varied widely. Only eight of the 24 unique mutations (Δ10A, 60A61, 96U97, 123A124, Δ180U, C259U, Δ300U, and Δ322G) were also found in the set of 64 unique unshared *de novo* mutations identified by dimer sequencing. Indels 123A124 and Δ180U, which were observed multiple times and in all three dimer sequencing experiments, are among this group. Nevertheless, that only eight of the 64 unique *de novo* mutations identified by dimer sequencing were represented in progeny populations suggests that most mutations are lost through selection.

**Table 3:** Mutation hotspots identified by deep sequencing progeny populations.

Hotspots	Library 1	Library 2	Total	Hotspots	Library 1	Library 2	Total
Δ10A	11	0	11	Δ180U	7	23	30
U18G	17	5	22	A182U	10	21	31
Δ53A	0	17	17	Δ233C	13	1	14
Δ60A	3	14	17	U238G	11	43	54
60A61	13	4	17	Δ251C	14	1	15
96U97	3	12	15	C259U	3	23	26
123A124	31	25	56	Δ300U	11	2	13
A135G	7	28	35	300A301	3	12	15
G138C	11	91	102	A310U	10	3	13
G146C	1	35	36	Δ322G	0	12	12
A150C	6	36	42	U329C	8	32	40
U161C	62	4	66	U331C	9	23	32

*N*. *benthamiana* plants were inoculated with PSTVd transcripts derived from a cloned master sequence, and full-length progeny genomes were sequenced. Mutations were identified in two independent libraries prepared from systemically infected leaves. The 24 mutations listed are those present in unique sequences with 10 or more reads per million in either or both libraries. Mutations highlighted in red were also found by dimer sequencing. The 731 total mutations include 499 base substitutions and 232 indels. See text for further details.

Other important differences likely related to selection were evident when *de novo* and progeny mutations were compared. However, we cannot formally rule out the possibility that some of the differences noted here are a result of bias attributable to the comparatively low depth of the dimer-sequencing data set. Of the 731 total mutations included in progeny analysis, nearly 70% were base substitutions (499/731), with indels comprising the remainder (232/731). By contrast, indels (56/102) outnumbered base substitutions (46/102) in the *de novo* mutations. Since frameshifts cannot occur in a non-coding RNA, this suggests that indels are more likely to have a detrimental impact on PSTVd RNA structure than substitutions. Among base substitutions, transversion events (300/499) were more frequent than transitions (199/499) in variant PSTVd progeny, while within *de novo* mutations transitions were more common. Similarly, among PSTVd progeny deletions (129/232) were slightly more common than insertions (103/232), while insertions and deletions were equally represented in the *de novo* mutation set. The impact of selection was also evident in the frequencies at which certain mutations were observed. It is perhaps not surprising that 123A124 and Δ180U constitute nearly 40% of total progeny indels (56+30/232) (Table 3), as these sites were hotspots for Pol II transcription errors. However, G138C and U238G, which were not identified by dimer sequencing, together account for more than more than half of transversion events (102+54/300). Likewise, U161C and U329C constitute more than half of total transitions (66+40/199). The abundance of certain variants compared to others suggests that the mutations they contain are less likely to have detrimental effects on PSTVd fitness. Extensive functional mutagenesis will be required to answer questions related to the fitness of individual mutations and differences between *de novo* and progeny mutation spectra.

Similarities also emerge from comparison of *de novo* and progeny mutations. First, with the exception of 96U97, Δ250C, and 300A301, indels observed in progeny populations occurred within homonucleotide tracts consisting of 3 to 6 residues. These include Δ10A, 60A61, 123A124, Δ180U, Δ300U, and Δ322G found in both data sets, as well as Δ53A, Δ60A, and Δ233C in PSTVd progeny (Fig 5B). Mutations observed in the progeny population were also present throughout the PSTVd genome and affected both stem and loop structures. Mutations were again clustered, although to a lesser extent, in sequences encompassing loop 9, and the 123A124 and Δ180U insertion and deletion hotspots were also observed. The cluster of substitutions in and around loops 3 and 4 seen by dimer sequencing, however, was mostly absent from PSTVd progeny. Finally, consistent with previous observations that gave rise to their names, the CCR and TCR domains constituting ~20% of the genome contained less than 9% (63/731) of total progeny mutations, including the 96U97 insertion and C259U substitution observed in both data sets.

In summary, similarities in mutation spectrum and landscape suggest that essentially all variants in PSTVd progeny populations ultimately arise from Pol II errors followed by selection.

## Conclusion

This study describes a novel (-)-stand dimer sequencing approach to assess the mutation rate and adaptation potential of PSTVd, a nuclear-replicating viroid. Using this method, which has the advantage of detecting the full spectrum of mutations across the entire genome in the absence of selection, we determined an apparent error rate for Pol II transcribing the PSTVd RNA genome that is ~100-fold greater than Pol II transcription on DNA templates (~5.4 x 10^−4^ per nucleotide per transcription cycle vs. ~4 x 10^−6^ per base pair). The error rate was used to calculate a PSTVd mutation rate (1/919 nucleotides for a single replication cycle) that is similar to estimates for chloroplast-replicating viroids. This very high mutation rate is likely sustainable given the small size of the PSTVd genome (359 nucleotides) and would allow abundant sampling of sequence space for adaptation to new environments and challenges. Further, the landscape of polymerase errors suggests that the most highly conserved genome regions, specifically the CCR and TCR, might reflect both critical function and greater Pol II fidelity, which in turn suggests co-evolution of PSTVd genome features and polymerase activity. High fidelity transcription in replication-critical genome regions coupled with an otherwise high mutation rate seems an optimal strategy to ensure viroid survival while maximizing adaptation potential. Finally, a comparison of mutations before selection (dimer dataset) and sequence populations after selection (deep sequencing dataset) allows us to propose that most polymerase errors are eliminated by strong host selection.

This work provides a basis for future studies of viroid and virus replication and evolution. The dimer-sequencing method can easily be applied to other pospiviroids, and perhaps also to members of the *Avsunviroidae*, although this may prove more challenging. It may also be useful for estimating mutation rates for viroid-like satellite RNAs and RNA viruses that employ rolling circle replication to generate greater than unit-length products, such as hepatitis delta virus [22,56].

## Materials and methods

### RNA preparation and plant inoculation

PSTVd RNA used to inoculate *Nicotiana benthamiana* plants was prepared as previously described [51]. A plasmid (pRZ6-2-Int) containing a T7 promoter and full-length cDNA of the PSTVd intermediate strain (PSTVd-I, GenBank accession number NC_002030) [57] was a gift of Dr. Robert Owens [58]. The plasmid was linearized with Hind III and transcription was carried out *in vitro* using T7 Megascript (ThermoFisher Scientific, Waltham, MA) to synthesize (+)-strand PSTVd RNA for plant inoculation. In a control experiment, a PCR product containing a T3 promoter and a PSTVd dimer was used as template to prepare *in vitro* transcripts with T3 Megascript (ThermoFisher Scientific). Following *in vitro* transcription, all reactions were treated with DNase I at 37° C for 1 h to completely remove template DNA. Transcripts were purified using the MEGAClear kit (ThermoFisher Scientific).

*N*. *benthamiana* plants were propagated in the Biotechnology Support Facility at The Ohio State University and inoculated as described [51]. Briefly, the first two true leaves of two-week old plants were first dusted with carborundum powder, followed by mechanical inoculation of (+)-PSTVd *in vitro* transcripts (300 ng/plant) in water treated with diethylpyrocarbonate. Three biological replicates were performed for each experiment, each including four plants.

### RNA extraction and reverse transcription

The upper three leaves of four plants in each biological replicate were collected at two- and three-weeks post inoculation and combined. RNA was isolated from plant tissues using the Quick-RNA Miniprep Kit (Zymo Research, Irvine, CA). Genomic DNA removal was performed using the same kit.

Beginning with 10 μg total RNA, SuperScript IV Reverse Transcriptase (ThermoFisher Scientific) was used to prepare cDNA from (-)-strand PSTVd RNA under conditions optimized for primer extension on the highly structured viroid RNA: 25°C for 10 min, 48°C for 15 min, 50°C for 15 min, 52°C for 20 min, and 80°C for 10 min. Two separate reactions were performed using two different primers: PSTVd1R (5'-CGGAACTAAACTCGTGGTTCC-3') and PSTVd2R (5'-GGCTACTACCCGGTGGAAAC-3').

### PCR amplification of (-)-strand PSTVd concatemers

To estimate the number of monomers included in (-)-strand PSTVd concatemers transcribed by Pol II, Taq DNA polymerase (GenScript, Piscataway, NJ), which has 5'→3' exonuclease activity, was used to perform PCR amplification with cDNA samples as template. It is important to note that cDNA was used in PCR reactions immediately after synthesis. Overnight storage at -80°C significantly reduced PCR productivity, sometimes resulting in production of only monomers. To maximize the likelihood of amplifying the largest PCR products, four PCR reactions were tested using the following primer pairs:

1. PSTVd1F (5'-AGGAACCAACTGCGGTTCC-3') + PSTVd1R (given above)
2. PSTVd1F* (5'-GAGGTCAGGTGTGAACCACA-3') + PSTVd1R
3. PSTVd2F (5'-GAAGCGACAGCGCAAAG-3') + PSTVd2R (given above)
4. PSTVd2F* (5'-GGTTCTCGGGAGCTTCAGTT-3') + PSTVd2R.

Primer sets were designed to amplify PCR products that are genome length or multiples of genome length. PCR reactions were performed under the following conditions: 95°C for 3 min, followed by 35 cycles of 95°C for 30 s, 56°C for 30 s, and 72°C for 2 min 50 s, with a final step at 72°C for 10 min. To determine the optimal elongation period (2 min 50 s), times ranging between 1 min and 5 min were tested at 10 s intervals. Shorter elongation times reduced production of longer PCR products, while elongation times greater than 2 min 50 s had no obvious effect on the outcome. Products were separated by agarose gel electrophoresis (1% in ice cold 0.5x TBE buffer) at 40 volts for 6 h and visualized by staining with ethidium bromide.

To obtain PCR products containing (-)-strand PSTVd dimers for sequencing, Phusion DNA Polymerase (NEB, Ipswich, MA), which has the lowest error rate of all commercially available DNA polymerases, was used to perform PCR reactions. Primer pair 2 was used in these experiments. PCR reactions were performed under the following conditions: 98°C for 1 min, followed by 28 cycles of 98°C for 10 s, 62°C for 10 s, and 72°C for 2 min 50 s, and finally 72°C for 10 min. The optimal elongation period (2 min 50 s) was determined as described above. PCR products were separated by agarose gel electrophoresis (1% in 0.5x TBE buffer) at 80 volts for 1 h and visualized by staining with ethidium bromide.

### Sequencing PSTVd dimers and identification of mutations shared or not shared by monomers

Taq DNA polymerase was employed to add poly(A) to the blunt ends of PSTVd PCR products amplified by Phusion polymerase (72°C for 30 min). Following gel electrophoresis, PSTVd dimer bands were excised and DNA was purified using the Zymoclean Gel DNA Recovery Kit (Zymo Research). DNAs were then ligated into a pCR2.1 vector and cloned using the Invitrogen TA Cloning Kit (ThermoFisher Scientific). After purification of recombinant plasmids, DNA inserts were subjected to Sanger sequencing at The Ohio State University Comprehensive Cancer Center. Sequences were analyzed using NCBI blast (https://blast.ncbi.nlm.nih.gov/Blast.cgi?PROGRAM=blastn&PAGE_TYPE=BlastSearch&LINK_LOC=blasthome), performed with dimer sequences as query and wild type PSTVd sequence as target.

### PSTVd quasispecies sequencing and data analysis

Library preparation, deep sequencing, and data analysis was described in a recently published study [55]. It should be noted that while different plants were used for library preparation and dimer sequencing, the same protocols were used to generate and deliver intermediate strain PSTVd inoculum RNA to *N*. *benthamiana* plants. Plants were propagated in growth rooms under the same conditions of light and temperature and inoculated at the same growth stage. Briefly, total RNA (10 μg) was isolated from pooled systemically infected leaves of *N*. *benthamiana* plants inoculated 14 and 21 days previously with wild type PSTVd. Progeny RNAs were reverse transcribed using a PSTVd-specific primer with Superscript IV Reverse Transcriptase (which has strand displacement activity) to generate greater than full-length cDNA from circular (+)-strand PSTVd genomic RNA. Full-length PSTVd (359 bp) was then amplified using Phusion DNA polymerase and sequenced using the MiSeq platform (Illumina, San Diego, CA). This technology allows sequencing of more than 250 nucleotides from each end, so that together paired end sequences spanned entire genome-length PSTVd cDNAs. Raw sequencing data, processed using a Python-based pipeline, was analyzed using Mothgur software as described [59]. Briefly, the 'make.contigs' function was used to build contigs after removal of adaptor and primer sequences. The 'screen.seqs' function was then used to remove sequences longer than desired and those containing ambiguous bases. The 'unique.seqs' and 'count.seqs' functions were employed to identify unique sequences in each dataset. To minimize errors resulting from library preparation, unique sequences with fewer than 30 reads per million were eliminated. Mutations shared by more than 10 unique sequences per one million reads above this threshold were compared with mutations identified by dimer sequencing analysis.
